# Supplementary material for: Caregiver Willingness to Vaccinate Children with Pneumococcal Vaccines and to Pay in a Low-Resource Setting in China: A Cross-Sectional Study
Source: Vaccines (Basel). 2022 Nov 10;10(11):1897. doi: 10.3390/vaccines10111897 (PMC9694362; doi:10.3390/vaccines10111897)
Supplement: Supplementary file 1 [file vaccines-10-01897-s001.zip › vaccines-1984993-supplementary.pdf]

Supplementary materials

Title: Caregiver willingness to vaccinate children with pneumococcal vaccines and willingness to pay in a low-resource setting in China: a cross-sectional study

Table S1. Behavioral and social factors related to vaccination

	**Total (N=1254)**	**Intention to get the child vaccinated with 13-valent pneumococcal conjugate vaccines (PCV13)**			***p***
		**Acceptance (N=899)**	**Hesitancy (N=254)**	**Refusal (N=101)**	
**Social and psychological context**					
**Baseline vaccine hesitancy**					
Perceived high importance, n (%)	1,168 (93.14)	864(96.11)	218(85.83)	86(85.15)	<0.001
Perceived high safety, n (%)	896 (71.45)	671(74.64)	153(60.24)	72(71.29)	<0.001
Perceived high effectiveness, n (%)	894 (71.29)	688(76.53)	150(59.06)	56(55.45)	<0.001
Willing to vaccinate their child with self-paid vaccines, n (%)	1,127 (89.87)	847(94.22)	210(82.68)	70(69.31)	<0.001
Vaccine preference, n (%)					0.002
Domestic	587 (46.81)	407(45.27)	123(48.43)	57(56.44)	
Imported	141 (11.24)	120(13.35)	19(7.48)	2(1.98)	
No preference	526 (42.95)	372(41.38)	112(44.09)	42(41.58)	
**Cues to action**					
The child has a history of pneumonia	190 (15.15)	145(16.13)	35(13.78)	10(9.90)	0.201
**Subjective norm**					
Religious concerns, n (%)	18 (1.44)	12(1.33)	5(1.97)	1(0.99)	0.699
The child's siblings have been vaccinated with PCV13, n (%)					<0.001
Yes	136 (10.85)	127(14.13)	5(1.97)	4(3.96)	

No or unclear	1,118 (89.15)	772(85.87)	249(98.03)	97(96.04)	
Family members have a medical background, n (%)	296 (23.60)	224 (24.92)	40 (15.75)	32 (31.68)	0.001
Have been recommended PCV13 by health workers, n (%)	306 (24.40)	261(29.03)	14(5.51)	31(30.69)	<0.001
Trust health workers' vaccination recommendation, n (%)	1,025 (81.74)	785(87.32)	173(68.11)	67(66.34)	<0.001
**Risk perception of pneumonia**					
Perceived high severity of pneumonia, n (%)	1,158 (92.34)	849(94.44)	230(90.55)	79(78.22)	<0.001
**Attitudes and beliefs of PCV13**					
Know that PCV13 is not the vaccine for COVID-19, n (%)	816 (65.07)	605(67.30)	134(52.76)	77(76.24)	<0.001
Know PCV13, n (%)	490 (39.07)	409(45.49)	37(14.57)	44(43.56)	<0.001
Know the price of PCV13, n (%)	301 (24.00)	261(29.03)	14(5.51)	26(25.74)	<0.001
Know the diseases that PCV13 can prevent, n (%)	126 (10.05)	105(11.68)	7(2.76)	14(13.86)	<0.001
PCV13 should be introduced to NIP, n (%)	1098 (87.56)	855(95.11)	174(68.50)	69(68.32)	<0.001

Note: NIP is the abbreviation for National Immunization Program.

Table S2. Intention to vaccinate children with 13-valent pneumococcal conjugate vaccines among participants of different characteristics

	Total (N=1254)	Intention to get the child vaccinated with 13-valent pneumococcal conjugate vaccines (PCV13)			*p*
		Acceptance (N=899)	Hesitancy (N=254)	Refusal (N=101)	
**Caregivers' characteristics**					
Age group (Years), n (%)					0.066
≤29	344(27.43)	255(74.13)	66(19.19)	23(6.69)	
30-34	492(39.23)	352(71.54)	99(20.12)	41(8.33)	
35-39	290(23.13)	214(73.79)	50(17.24)	26(8.97)	
≥40	128(10.21)	78(60.94)	39(30.47)	11(8.59)	
Sex, n (%)					0.005*
Male	288(22.97)	200(69.44)	74(25.69)	14(4.86)	
Female	966(77.03)	699(72.36)	180(18.63)	87(9.01)	
Educational attainment, n (%)					<0.001*
High school and below	488(38.92)	314(64.34)	127(26.02)	47(9.63)	
Bachelor's degree and above	766(61.08)	585(76.37)	127(16.58)	54(7.05)	
Relationship to the child, n (%)					0.004*
Mother	950(75.76)	691(72.74)	174(18.32)	85(8.95)	
Father	284(22.65)	198(69.72)	72(25.35)	14(4.93)	
Grandparent	20(1.59)	10(50.00)	8(40.00)	2(10.00)	
Annual household income (10 thousand CNY), n (%)					0.055
<5	378(30.14)	249(65.87)	93(24.60)	36(9.52)	
5-15	597(47.61)	433(72.53)	116(19.43)	48(8.04)	
15-25	195(15.55)	149(76.41)	33(16.92)	13(6.67)	
≥25	84(6.70)	68(80.95)	12(14.29)	4(4.76)	
**Children's characteristics**					
Sex, n (%)					0.351
Male	628(50.12)	441(70.22)	130(20.70)	57(9.08)	
Female	625(49.88)	457(73.12)	124(19.84)	44(7.04)	
Age group (Months), n (%)					0.121
<6	393(31.34)	292(74.30)	70(17.81)	31(7.89)	
6-11	362(28.87)	265(73.20)	76(20.99)	21(5.80)	
12-23	271(21.61)	193(71.22)	51(18.82)	27(9.96)	
24-59	228(18.18)	149(65.35)	57(25.00)	22(9.65)	
An only child, n (%)	612 (48.80)	473(77.29)	107(17.48)	32(5.23)	<0.001*
**Social and psychological context**					

**Baseline vaccine hesitancy**					
Perceived high importance, n (%)	1,168 (93.14)	864(73.97)	218(18.66)	86(7.36)	<0.001*
Perceived high safety, n (%)	896(71.45)	671(74.89)	153(17.08)	72(8.04)	<0.001*
Perceived high effectiveness, n (%)	894 (71.29)	688(76.96)	150(16.78)	56(6.26)	<0.001*
Willing to vaccinate their child with self-paid vaccines, n (%)	1,127 (89.87)	847(75.16)	210(18.63)	70(6.21)	<0.001*
Vaccine preference, n (%)					0.002*
Domestic	587(46.81)	407(69.34)	123(20.95)	57(9.71)	
Imported	141(11.24)	120(85.11)	19(13.48)	2(1.42)	
No preference	526(41.95)	372(70.72)	112(21.29)	42(7.98)	
**Cues to action**					
The child has a history of pneumonia	190 (15.15)	145(76.32)	35(18.42)	10(5.26)	0.201
**Subjective norm**					
Religious concerns, n (%)	18 (1.44)	12(66.67)	5(27.78)	1(5.56)	0.699
The child's siblings have been vaccinated with PCV13, n (%)					<0.001*
Yes	136 (10.85)	127(93.38)	5(3.68)	4(2.94)	
No or unclear	1,118 (89.15)	772(69.05)	249(22.27)	97(8.68)	
Family members have a medical background, n (%)	296 (23.60)	224(75.68)	40(13.51)	32(10.81)	0.001*
Have been recommended PCV13 by health workers, n (%)	306 (24.40)	261(85.29)	14(4.58)	31(10.13)	<0.001*
Trust health workers' vaccination recommendation, n (%)	1,025 (81.74)	785(76.59)	173(16.88)	67(6.54)	<0.001*
	**Risk perception of pneumonia**				
Perceived high severity of pneumonia, n (%)	1,158 (92.34)	849(73.32)	230(19.86)	79(6.82)	<0.001*
	**Attitudes and beliefs of PCV13**				
Know that PCV13 is not the vaccine for COVID-19, n (%)	816 (65.07)	605(74.14)	134(16.42)	77(9.44)	<0.001*
Know PCV13, n (%)	490 (39.07)	409(83.47)	37(7.55)	44(8.98)	<0.001*
Know the price of PCV13, n (%)	301 (24.00)	261(86.71)	14(4.65)	26(8.64)	<0.001*
Know the diseases that PCV13 can prevent, n (%)	126 (10.05)	105(83.33)	7(5.56)	14(11.11)	<0.001*

PCV13 should be introduced to NIP, n (%)	1098 (87.56)	855(77.87)	174(15.85)	69(6.28)	<0.001*

**p*<0.05.

Table S3. Sources for knowledge about 13-valent pneumococcal conjugate vaccines

**Sources**	**Number of participants (N=490)**	**Percent (%)**
Publicity and education efforts in communities or hospitals	215	43.88
Internet (QQ, Wechat, Microblog, News websites)	183	37.35
Friends and relatives	53	10.82
Television, radio, newspapers	35	7.14
Others	4	0.82

Table S4. Reasons for refusal and hesitancy about getting children vaccinated with 13-valent pneumococcal conjugate vaccines

Reasons	Total, n (%)	Hesitancy, n (%)	Refusal, n (%)	*p*
**Thinking and feeling**				
Insufficient vaccine knowledge	223 (62.82)	191 (75.20)	32 (31.68)	<0.001
Uncertain about effectiveness	74 (20.85)	56 (22.05)	18 (17.82)	0.377
Uncertain about safety	69 (19.44)	55 (21.65)	14 (13.86)	0.094
Non-NIP vaccine	59 (16.62)	33 (12.99)	26 (25.74)	0.003
Low disease risk	25 (7.04)	8 (3.15)	17 (16.83)	<0.001
**Practical issues**				
Cost concerns	90 (25.35)	56 (22.05)	34 (33.66)	0.023
Inconvenient time	10 (2.82)	7 (2.76)	3 (2.97)	1.00
Unclear about vaccination site	6 (1.69)	5 (1.97)	1 (0.99)	0.850
**Others**				
Contraindication	6 (1.69)	5 (1.97)	1 (0.99)	0.850
Others	7 (1.97)	3 (1.18)	4 (3.96)	0.202

Note: Chi-square test was used to make a comparison between hesitancy and refusal groups. Non-NIP vaccine indicates non-national immunization program vaccine.

Table S5. Willingness to pay for 13-valent pneumococcal conjugate vaccines excluding those with vaccine refusal

	**Number of participants, N (%)**	**Willingness to pay (CNY)**
Total	1,153 (100.00)	-
Payment schemes		
Full payment (price uninformed)	813 (70.51)	-
Full payment (price informed)	558 (48.40)	2,588.00
Partial payment	477 (41.37)	920.00 (553.00-1,512.00)
Only service charge	91 (7.89)	88.00
Completely free	12 (1.04)	0.00
Others	15 (1.30)	-

Note: The expenses for full payment, service charge only, and completely free are fixed values. The expense for partial payment is represented
